# Aqueous two-phase system patterning of detection antibody solutions for cross-reaction-free multiplex ELISA

**DOI:** 10.1038/srep04878

**Published:** 2014-05-02

**Authors:** John P. Frampton, Joshua B. White, Arlyne B. Simon, Michael Tsuei, Sophie Paczesny, Shuichi Takayama

**Affiliations:** 1 Department of Biomedical Engineering, University of Michigan; 2 Department of Macromolecular Science and Engineering, University of Michigan; 3 Department of Pediatrics, Indiana University School of Medicine

## Abstract

Accurate disease diagnosis, patient stratification and biomarker validation require the analysis of multiple biomarkers. This paper describes cross-reactivity-free multiplexing of enzyme-linked immunosorbent assays (ELISAs) using aqueous two-phase systems (ATPSs) to confine detection antibodies at specific locations in fully aqueous environments. Antibody cross-reactions are eliminated because the detection antibody solutions are co-localized only to corresponding surface-immobilized capture antibody spots. This multiplexing technique is validated using plasma samples from allogeneic bone marrow recipients. Patients with acute graft versus host disease (GVHD), a common and serious condition associated with allogeneic bone marrow transplantation, display higher mean concentrations for four multiplexed biomarkers (HGF, elafin, ST2 and TNFR1) relative to healthy donors and transplant patients without GVHD. The antibody co-localization capability of this technology is particularly useful when using inherently cross-reactive reagents such as polyclonal antibodies, although monoclonal antibody cross-reactivity can also be reduced. Because ATPS-ELISA adapts readily available antibody reagents, plate materials and detection instruments, it should be easily transferable into other research and clinical settings.

The enzyme-linked immunosorbent assay (ELISA) is used in clinical and laboratory settings to accurately and reproducibly quantify soluble proteins^1^. ELISA is typically performed in a sandwich format by immobilizing capture antibodies on an assay plate, allowing specific antigens to associate with the surface immobilized antibodies, and then detecting the antigens by way of detection antibodies that generate chromogenic, fluorescent or chemiluminescent signals^2^,^3^.

Owing to their versatility and reliability, ELISAs have been used to detect HIV/AIDS^4^, malaria^5^, cancer^6^,^7^ and inflammatory/autoimmune diseases^8^,^9^, among numerous other pathologies. However, conventional singleplex ELISA formats are limited by high reagent costs, inefficient use of patient samples and an inability to prevent antibody cross-reactions when multiplexed^10^,^11^. For example, multiplex platforms that spatially segregate capture antibodies to many individual spots or beads within an assay-well can greatly increase ELISA throughput; however, this advantage is often undercut by problems associated with cross-reactions among detection antibodies applied as a cocktail^12^,^13^. The interactions among detection antibodies or between detection antibodies and inappropriate capture antibodies or antigens can lead to false-positive or false-negative readouts. The chance of some form of cross-reactivity greatly increases with each new detection antibody added to a multiplex panel, especially when the antibodies are polyclonal, as is the case for the majority of commercially available ELISA kits.

Many diseases, for example acute graft versus host disease (GVHD), cannot be diagnosed with sufficient specificity and sensitivity using single biomarkers^14^. Acute GVHD occurs in approximately half of allogeneic bone marrow transplantation recipients when donor immune cells recognize the host tissues as foreign and attack them. This reaction can be minimized by carefully matching the donor and host tissues and using prophylactic immunosuppression, but it is still the leading cause of non-relapse mortality in this population. Pre-transplant clinical or transplant characteristics have minimal ability to predict acute GVHD outcomes. Currently, acute GVHD is diagnosed by clinical symptoms in three organ systems (skin, liver and gastrointestinal tract) and may be confirmed using biopsies. Therefore, at the time of diagnosis, patients can already have substantial organ damage. Recently, there has been a push to develop multi-biomarker immunoassays for conclusive acute GVHD diagnosis before the onset of symptoms, because in the event that a patient develops acute GVHD, it is critically important to treat them early to prevent organ damage^8^,^9^,^14^,^15^,^16^. Unfortunately, as explained above, it is difficult to develop and implement multi-biomarker panels for clinical settings because of cross-reactions among antibodies that complicate the multiplexed validation of new biomarkers due to false readouts. Misdiagnosis of acute GVHD can be particularly dangerous to patients since immunosuppressive treatments themselves can result in sepsis and early malignancy relapse via loss of the graft-versus-tumor effect.

Many multiplex assays rely on the application of a cocktail of detection antibodies. After extensive optimization, such cocktails can often provide satisfactory results^17^,^18^,^19^,^20^,^21^. However, this type of optimization can be costly and time consuming. Moreover, if additional biomarkers are added to the panel or the antibody reagents change, the process of optimization must be repeated. To avoid this problem, we previously resorted to performing serial singleplex ELISAs^22^.

Previously, several other approaches were developed to mitigate antibody cross-reactions for improved multiplex protein detection^23^,^24^,^25^. For example, antibody colocalization microarrays use aligned spots of capture and detection antibodies that are dispensed in microliter volumes in air on nitrocellulose membranes. However, there is a tendency for the antibody solutions to evaporate under ambient conditions, leading to antibody and biomarker degradation. Bead-based assays, such as Luminex^TM^, can theoretically achieve up to 100-plex. In reality, however, capture and detection antibody cross-reactions limit Luminex^TM^-based protein detection to the initial screening of biomarkers, rather than clinical biomarker verification^26^. Finally, advanced array printing technologies, such as continuous flow print heads^27^,^28^, allow aligned deposition of multiple protein solutions (including antibodies and sample solutions) with limited evaporation, but require specialized, non-standard laboratory equipment that may be difficult to access by laboratories and clinics.

Here, we eliminate the problem of detection antibody cross-reactions in multiplex ELISA by using aqueous two-phase systems (ATPSs) composed of the phase-separation-promoting polymers polyethylene glycol (PEG) and dextran (DEX)^27^,^28^ to confine detection antibody solutions in a fully aqueous environment to regions where complementary capture antibodies are immobilized^29^,^30^. This method, referred to as ATPS-ELISA, works on three principles: i. droplets of the denser DEX solution sink in the PEG solution and remain in contact with the assay plate during incubation; ii. interfacial tensions between DEX-PEG and DEX-assay plate cause the DEX droplets to form domes that remain in place; and iii. detection antibodies are retained, without diffusive dispersion, in the DEX phase due to partitioning effects. We demonstrate the translational potential of this technology by simultaneously detecting at least four different antigens that were previously demonstrated to be associated with acute GVHD, a disease that requires multiple biomarker analysis for definitive diagnosis and prognosis^14^. Our assay is not only free from antibody cross-reactions, but also requires less antibody and patient sample than conventional single biomarker ELISA.

## Results

### Detection Antibody Micro-Droplet Patterning Prevents Cross-Reactions

**Figure 1:**
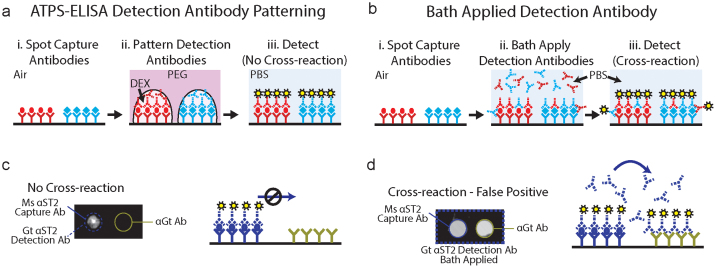
ATPS-ELISA prevents polyclonal detection antibody cross-reactions. Multiplex ATPS-ELISA (a) and conventional sandwich ELISA (b) share similar procedures, shown in steps i., ii., and iii. However, by co-localizing detection antibodies (dashed line antibody symbols) in the DEX phase over the corresponding capture antibodies (solid line antibody symbols) through the use of simple micropipetting onto custom plates with features designed for micropipette tip alignment, ATPS-ELISA produces signals without any possibility of cross-reactions. (c) Goat detection antibodies were not captured by a neighboring spot coated with an anti-goat capture antibody, indicating that antibodies did not diffuse out of the DEX droplet to cause cross-reactions. (d) Bath application of detection antibodies resulted in cross-reactions at the neighboring capture antibody spot.

**Figure 2:**
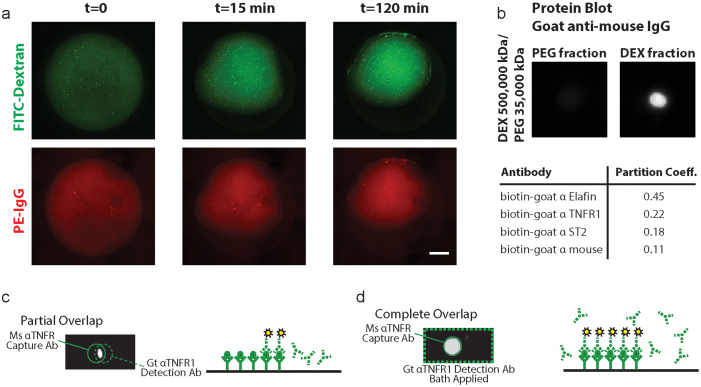
DEX droplets assume dome shapes that change very little in size and shape over the course of the incubation period. (a) Antibodies are retained in the DEX domes over this period as indicated by the overlap between FITC-DEX and PE-IgG. Scale bar = 1 mm. (b) Biotin-labeled ELISA detection antibodies partition favorable to the DEX phase. Partition coefficients were measured by blotting detection antibody fractions from PEG and DEX on PVDF membranes and detecting the antibody levels by way of streptavidin-HRP chemiluminescence. Partitioning can be further improved by modifying ATPS formulations. (c) Partial overlap of the capture and detection antibodies results in a cat eye shape that can only be produced if antibodies are well retained in the DEX droplet. (d) Bath application of detection antibodies produced a circular signal area.

ATPS-ELISA is performed using the same steps as conventional sandwich ELISA, except that ATPS-ELISA uses detection antibody solutions that are deposited in DEX droplets over the capture antibody spots (Figure 1 a and b) by micropipetting. This prevents cross-reactions between unwanted pairs of polyclonal capture and detection antibodies, as well as from non-target antigen recognition by detection antibodies. The capture antibodies and detection antibodies can be easily aligned using polystyrene plates with embossed features (Figure S1), although it is also possible to perform the assay using planar polystyrene substrates, as shown in Figures 1 and 2. Figure 1 c shows the effectiveness of ATPS-ELISA at eliminating antibody cross-reactions. We intentionally spotted polyclonal antibodies that are more prone to cross-react. One spot contained an immobilized anti-human ST2 capture antibody, while the adjacent spot contained an immobilized anti-goat antibody that recognized the detection antibody (a goat anti-human ST2 antibody). After incubating with a solution containing ST2, the goat detection antibody was either deposited above the mouse capture antibody-antigen complex in DEX using an ATPS or bath applied in the conventional manner without using ATPS (Figure 1 c, d). There was no cross-reactive signal for the ATPS format (Figure 1 c), demonstrating that the detection antibodies remained confined to DEX and were unable to diffuse away to react with the anti-goat antibody spot. On the other hand, conventional bath application allowed detection antibodies to freely circulate, resulting in strong signals at both the appropriate capture antibody spot and the spot containing the anti-goat antibody (Figure 1 d). This illustrates the possibility of false-positive readouts that may lead to misinterpretation of the biomarker panel when inappropriate combinations of detection antibodies are used in conventional multiplex systems.

As shown in Figure 2 a, phycoerythrin (PE)-IgG was well retained within a DEX droplet over the course of 2 h. Furthermore, analysis of the distributions (i.e., the partition coefficients) of the detection antibodies within equilibrated ATPSs indicated that the antibodies partitioned predominantly to DEX (Figure 2 b). The cat eye-shaped signal in Figure 2 c shows that ATPS-ELISA can localize the detection antibodies to specific regions and that signal is restricted only to the regions where both the detection antibody solution droplet and the surface-adsorbed capture antibody spot overlap. In Figure 2 d, the detection antibody was bath applied, producing a larger circular signal spot because the detection antibody solution covered the entire capture antibody spot. These results demonstrate the ability of ATPS-ELISA to suppress detection antibody cross-reactions, while highlighting the need to align the detection antibody solution droplets over the capture antibody spots to avoid signal reducing artifacts.

The impact of optical crosstalk was not significant in our system, as assessed by signal area intensity plots and background measurements (Figure S2). These plots also demonstrated that the signal intensities of the sample regions were relatively uniform for both high and low antigen concentrations using ATPS detection antibody spotting.

### ATPS-ELISA Facilitates Multiplex GVHD Biomarker Detection

**Figure 3:**
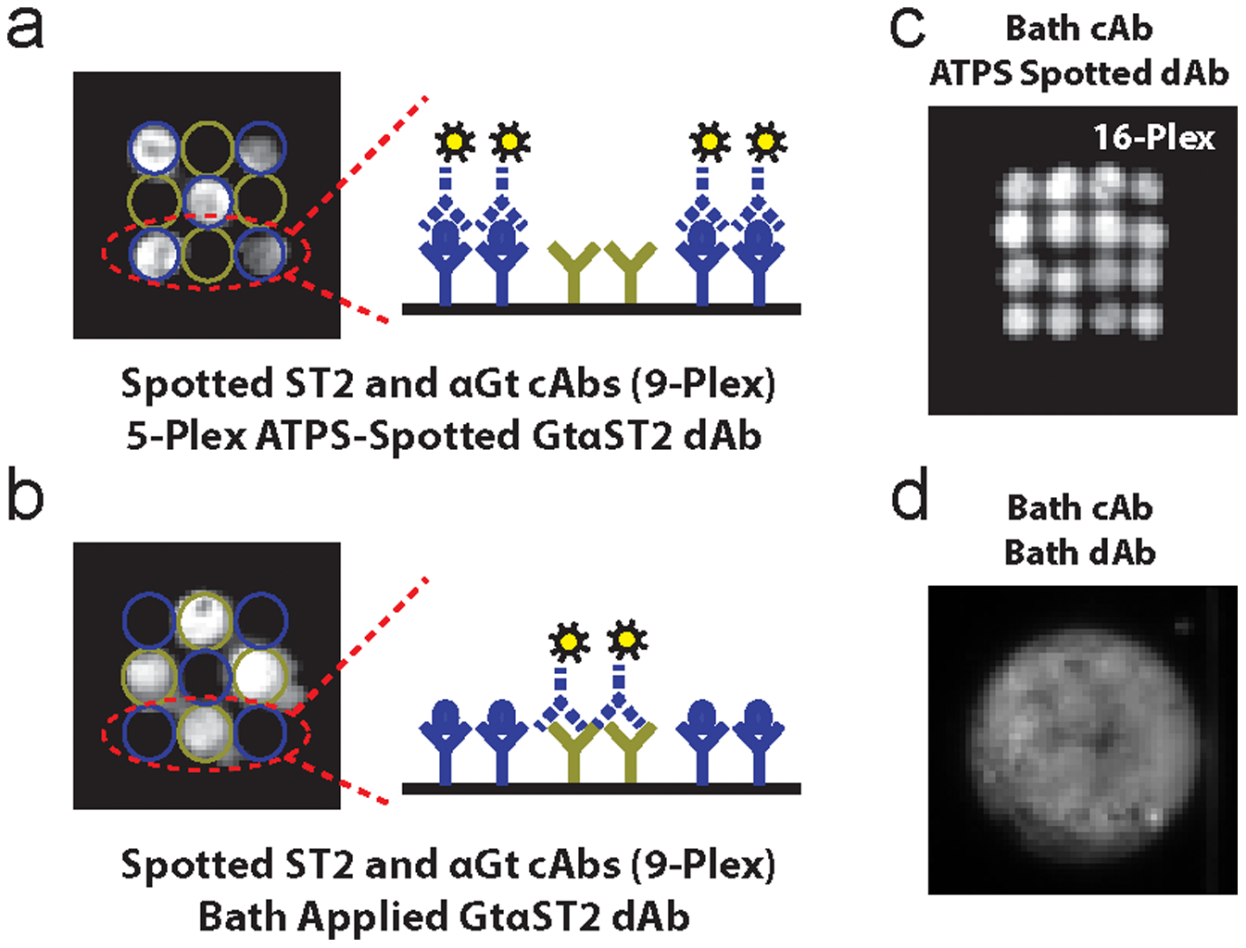
ATPS ELISA can be used to pattern 9-plex and 16-plex arrays of detection antibodies. (a) A 9-plex version of the experiment shown in Figure 1 c that demonstrates how ATPS-ELISA suppresses cross-reactions. Goat anti-ST2 detection antibody solutions are dispensed as ATPS droplets only to the 5 regions with spotted ST2 capture antibodies and ST2 antigen. These 5 regions generated true-positive signals. The 4 other regions spotted with anti-goat capture antibodies that would cross-react and give false positive signals with the goat anti-ST2 detection antibody did not receive any detection antibody solution droplets and thus resulted in no signal, demonstrating suppression of false positive signals. (b) A 9-plex version of the experiment shown in Figure 1 d. Bath applied goat anti-ST2 detection antibodies become sequestered by the 4 anti-goat antibody spots. This produces 4 false positive readouts and interestingly 5 false negative signals with very low (but detectable) signal levels because there is insufficient ST2 detection antibody available to bind to the ST2 sandwich regions despite the fact that the anti-ST2 capture antibodies are bound to ST2. (c) Representative image of a 16-plex ATPS sandwich ELISA for ST2 with bath application of the capture antibody and localized dispensing of detection antibody in DEX droplets. (d) Same experiment as in c but without localized dispensing of detection antibody in the DEX droplets. The result is a signal from the entire well rather than localized signals.

Custom plates molded from polystyrene were used to facilitate capture antibody and detection antibody alignment in our multiplex assays. The plates were designed to prevent movement of the DEX droplets after pipetting and to prevent inadvertent misalignment of the capture and detection antibody spots. Prior to fabricating our final plate, we tested a range of antibody well diameters (0.5 mm to 2 mm) with different edge-to-edge spacing (0.3 mm to 0.6 mm) and a depth of ~110 µm. Using the smallest well diameter and spacing, it was possible to deposit a 7 × 7 array (49-plex) of detection antibodies, as well as 4 × 4 (16-plex) and 3 × 3 (9-plex) detection antibody arrays (Figure 3). Figure 3 a, b shows an example of a 9-plex array with an experimental setup similar to Figure 1 c, d. When applied in DEX, the goat anti-ST2 detection antibodies correctly localize to the 5 spots in the 9-plex array where the anti-ST2 capture antibodies are located with bound ST2 protein (Figure 3 a). However, the greater number of anti-goat capture antibody spots (4 spots as opposed to the single spots in Figure 1 d; i.e., approximately 4 times the area of spotted capture antibody) serve to sequester the goat-anti-ST2 detection antibodies when they are bath applied in a 10 µL volume, producing false negative readouts at the locations where the ST2 capture antibodies are localized and false positive readouts where the anti-goat antibodies are localized (Figure 3 b). Figure 3 c shows an example of 16-plex ST2 detection antibody patterning for a bath applied ST2 capture antibody. Bath application of the detection antibody results in chemiluminescent signal throughout the entire sample well (Figure 3 d).

**Figure 4:**
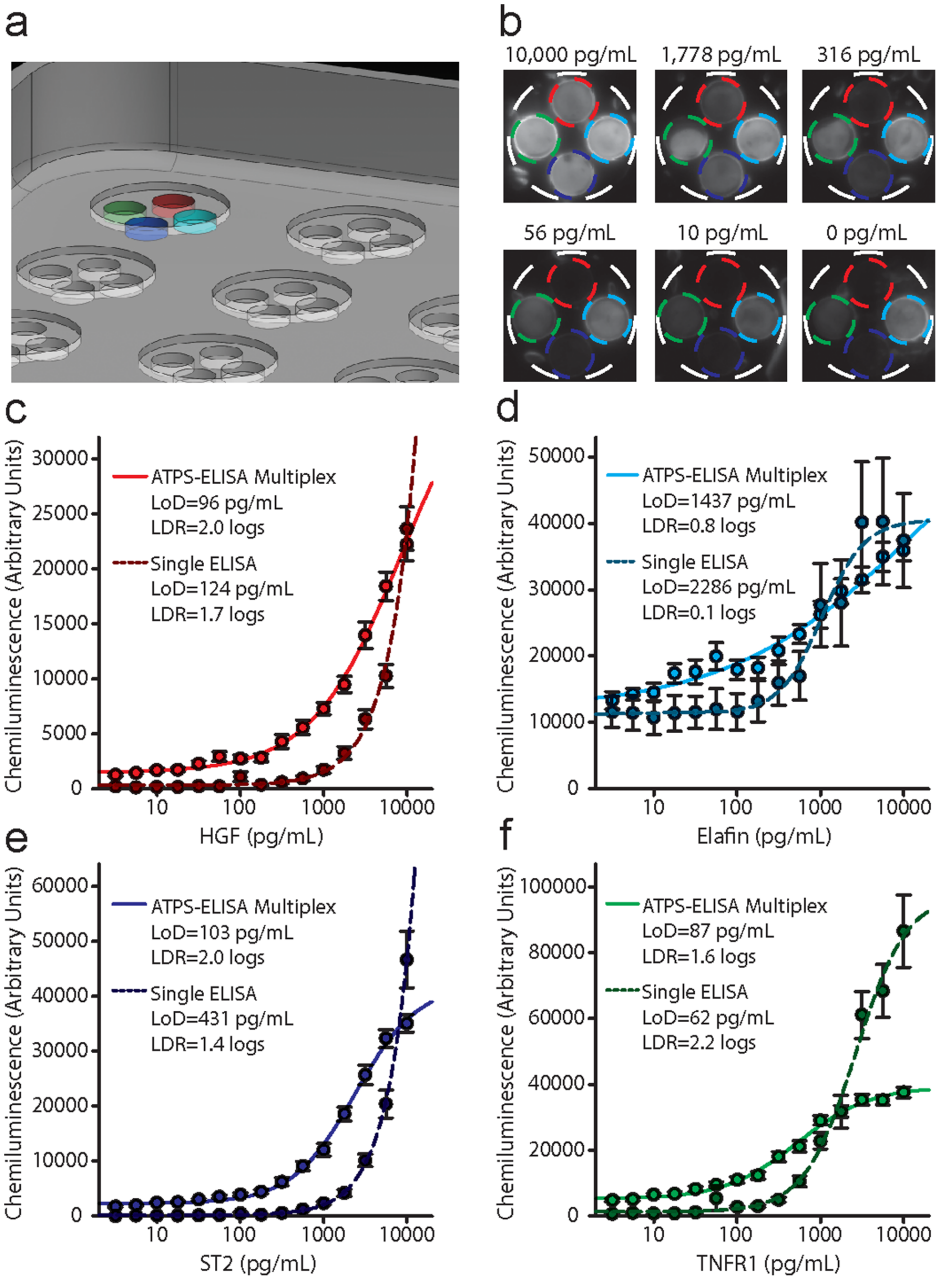
Multiplex ATPS-ELISA for GVHD biomarkers. (a) A 3D rendering showing the custom plate design consisting of 4 antibody insets within a common shallow sample well. (b) Representative images of the chemiluminescent standards. The concentrations listed above each image are the same for each biomarker in the panel. The images correspond to every third point on the quarter-logarithmic dilution curve from 10,000 pg/mL to zero. In each of the images the spot at the top corresponds to HGF, the spot on the right corresponds to elafin, the spot at the bottom corresponds to ST2 and the spot on the left corresponds to TNFR1. It is apparent from these images that elafin has the highest limit of detection due to its high background (elafin can be detected in healthy plasma as well as in GVHD+ plasma). Since the biomarker standards contained a 10% healthy pooled plasma to adjust for matrix affects (see “Plasma samples” in the Methods section), this background can be attributed to baselines levels of elafin and should not be confused as an example of a false positive signal. (c–e) Standard curves for all four GVHD biomarkers in PBS generated by densitometric quantification of chemiluminescence images compared to individual sandwich ELISAs (dashed lines). Error bars represent standard error of the mean.

Based on the number of biomarkers selected for our study, we designed our final plate with four antibody wells (4-plex; Figure 4 a) that were 1.5 mm in depth. These antibody wells functioned to hold the capture and detection antibody solutions in place during plate manipulation and transport. They also provided visual and tactile cues to ensure that we could easily perfect the alignment of the capture and detection antibody solutions using a multi-pipettor. Standard curves for 4 acute GVHD biomarkers (HGF, elafin, ST2 and TNFR1) were generated using the optimized antibody conditions suggested by the manufacturer for both multiplex ATPS-ELISA and individual ELISA. The limits of detection and linear dynamic range (LDR) values for ATPS-ELISA were generally comparable to individual sandwich ELISAs (Figure 4 b–f), although in some cases (e.g., ST2 and TNFR1), the standard curves for ATPS-ELISA reached saturation slightly before the standard curves for individual sandwich ELISA. The LoD and LDR values were also acceptable for analysis of acute GVHD patient samples.

### Validation of the Multiplex GVHD Assay

**Figure 5:**
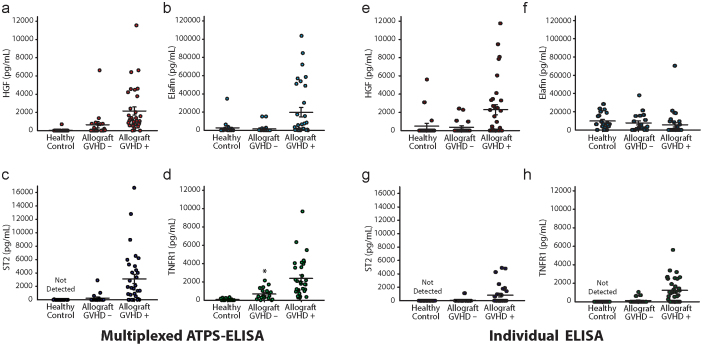
Multiplex ATPS-ELISA enables robust detection of GVHD biomarkers in patient plasma samples. The ATPS-ELISA multiplex detection system was used to probe human plasma for four biomarkers: (a) HGF, (b) elafin, (c) ST2 and (d) TNFR1. The GVHD+ group displayed significantly higher levels of all four biomarkers compared to the GVHD – and healthy control groups (p<0.05 by one-way ANOVA with Dunn's multiple comparison test). Measurements of (e) HGF, (f) elafin, (g) ST2 and (h) TNFR1 from patient plasma using individual sandwich ELISAs are provided for comparisons. Significance between the GVHD+ group and the GVHD- and healthy control groups was obtained for HGF and TNFR1 (p<0.05 by one-way ANOVA with Dunn's multiple comparison test) for individual ELISAs, but not for elafin or ST2, although ST2 values for the GVHD+ group tended to be higher than the other groups. Error bars represent standard error of the mean.

We analyzed plasma samples from three patient groups: healthy controls (n = 20), allogeneic bone marrow transplant patients who did not manifest symptoms of acute GVHD (GVHD –; n = 19, median 28 days post-transplant) and allogeneic bone marrow transplant patients who had been diagnosed with acute GVHD (GVHD+; n = 32, median 28 days post-transplant). The biomarkers and samples we used were used previously as part of several larger studies that analyzed GVHD status using serial singleplex ELISA^9^,^14^,^16^,^22^. We were blind to the GVHD status of the samples during the experiment and the image quantification. As expected, the GVHD+ patient group had significantly higher levels of HGF, elafin, ST2 and TNFR1 than the GVHD – group and the healthy donor group (Figure 5 a–d), demonstrating the effectiveness of our system at measuring multiple biomarkers from patient samples.

The ATPS-ELISA procedure, allowed us to accurately assess the GVHD status of the samples (Figure 5 e–h). For example, ATPS was slightly more effective at discriminating elafin levels between GVHD− and GVHD+ patient samples, because the higher level of elafin background produced more inconsistent readings in well plates compared to the low-profile ATPS-ELISA wells, possibly due to differences in the plate geometries. For example, due to the lower surface area of the capture antibody-coated regions in the ATPS-ELISA wells (~1.77 mm^2^), we would expect there to be greater signal per unit volume (10 µL) of antigen compared to the 384-well plate reservoirs used for single ELISA (>23 mm^2^). This is reflected in our standard curve data, which show that the ATPS-ELISAs enter linear ranges for signal detection at lower antigen concentrations than the conventional single ELISAs. These observations are consistent with the observations of others that suggest that smaller spot sizes relative to the assay volumes produce superior LoD and LDR values^31^,^32^. It is also possible that ATPS-ELISA benefits from microscale surface localization effects that enhance detection antibody binding, more effective washing due to the shallow well profiles and less depletion of chemiluminescent substrate, all of which can affect LoD and LDR. In addition, we note that the elafin ELISA reagents tended to be less robust and expire more quickly than the reagents from the other kits, which may explain why the elafin standard curves displayed higher limits of detection and lower linear dynamic ranges than the other biomarkers in the panel.

**Figure 6:**
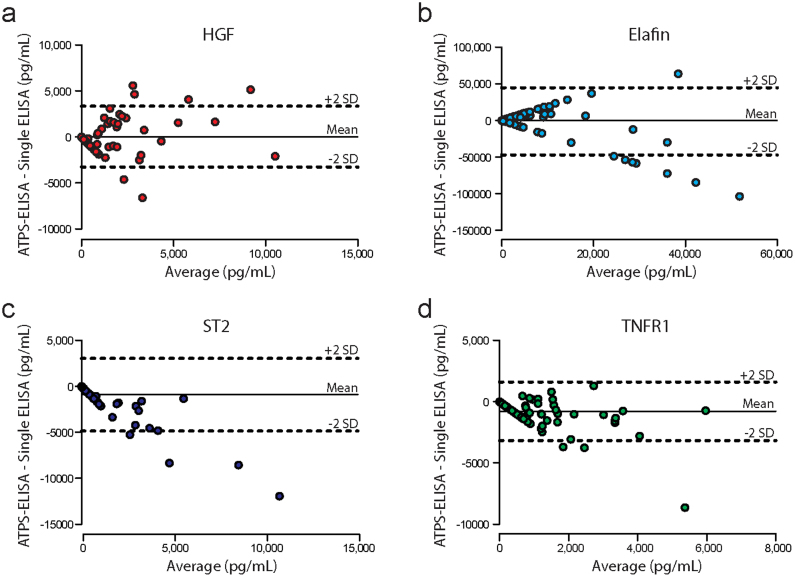
Bland-Altman analysis for (a) HGF, (b) elafin, (c) ST2 and (d) TNFR1. The dashed horizontal lines represent the 2SD confidence intervals and the solid horizontal line represents the mean difference between assay formats. We observed discrepancies between the two assay formats, particularly at the higher biomarker concentrations.

Bland-Altman analysis was used to evaluate the agreement between ATPS-ELISA and standard single ELISA (Figure 6). We observed proportional and magnitude biases between the ATPS-ELISA and single ELISA formats, indicating that there were discrepancies between the two methods. These discrepancies were generally tolerable at lower biomarker concentrations, for example, below 2,500 pg/mL for HGF, 36,000 pg/mL for elafin, 4,700 pg/mL for ST2 and 1,800 pg/mL forTNFR1 (i.e., concentrations that approximate the clinically important thresholds for GVHD diagnosis). Although a larger patient cohort would be required to determine clinical cutoffs for our assay, the approximate clinical cutoffs can be obtained from previous biomarker studies for HGF^33^, elafin^9^, ST2^16^ and TNFR1^14^. It is important to note that although the Bland-Altman analyses indicated discrepancies between the two assay formats, this does not necessarily indicate that one format is superior to the other. To provide a comparison of the performance of the two methods, we conducted receiver operating characteristic (ROC) analysis (Figure S3). In our hands, the ATPS format slightly outperformed the single ELISA format, as assessed by the areas under the curves, indicating that the ATPS method might provide superior sensitivity and specificity. Larger experiments involving greater numbers of biomarkers and additional samples, along with experimenter-to-experimenter comparisons, will be required to conclusively prove this assertion. However, these experiments are beyond the scope of the present study, which focuses on the development of a promising new technology for detection antibody patterning.

It is also important to emphasize that the multiplex biomarker panel was critical for obtaining high sensitivity (correct classification of patients with GVHD) and high specificity (correct classification of patients without the disease). For example, one of the GVHD– patients had high levels of HGF (6615 pg/mL). Based only on the levels of this biomarker, this patient could have been diagnosed with acute GVHD and could have potentially received unnecessary treatment. However, with the inclusion of the three other markers measurements that were all low, this patient can be considered GVHD– and avoid unnecessary treatments that may cause dangerous side effects. In other cases, a single biomarker did not reach the diagnostic threshold for GVHD in spite of the patient's GVHD+ status. In these cases the information obtained from the other three biomarkers in the panel that are all elevated can be used to correctly assess the disease status of the patient.

## Discussion

We demonstrated that our system can utilize chemiluminescence to determine the levels of biomarkers without antibody cross-reactions that can cause false positive and false negative signals using heavily cross-reacting antibodies as a test example (Figures 1, 2 and 3). We further validated the usefulness of the ATPS-ELISA technology by multiplexing 4 GVHD biomarkers that previously had to be measured using sequential single ELISA^14^,^22^. Although this is a relatively small panel, 4-plex represents a threshold at which running single ELISAs begins to become prohibitive in terms of assay time, material costs and sample consumption, especially when using precious samples where serial single ELISAs must be performed^14^,^22^. Furthermore, the working principles of the assay ensures that the degree of plexing is not limited by increasing concentrations of antibody cocktail solutions, since each detection antibody reagent solution is confined by the ATPS only to its corresponding capture antibody spot.

Our assay offers several advantages over conventional single biomarker ELISAs including the use of small plasma volumes (<10 µL total of diluted plasma for 4 biomarkers and <1 µL of patient sample per well), cost savings (less antibody consumption) and use of readily available plate materials and detection systems. For example, performing similar experiments using the individual wells of a conventional 384-well plate would require at least 40 µL of sample (~10 µL/well) to fill the 4 wells required for each of the 4-biomarkers tested. In contrast, our detection antibody patterning technology uses only 10 µL of sample total, since the 4 antibody spots are patterned within a low profile well of 6.5 mm diameter (an intermediate diameter between 96 and 384 format wells). We used 384 well format plates for our comparison to singleplex ELISA. Although the smaller well size allowed us to use 10 µL sample volumes for both the ATPS and regular ELISA assay formats, we did observe higher background and slightly worse ROC when using 384 well plates, likely due to a difference in the capture antibody area to sample volume ratio and possibly also due to the deep well geometry of conventional 384 well plates that can increase pipetting errors and make it more difficult to wash completely.

The ATPS-ELISA also provides advantages over miniaturized single ELISA systems, such as Droparray, which use non-aqueous solutions (i.e., oils) to cover the aqueous droplets^34^. Droparray utilizes droplet sizes (~2 µL) on the same order as the DEX droplets used in our ATPS-ELISA. However, Droparray is not designed as a multiplex immunoassay, since, in a similar fashion to a 384-well plate assay, the reagents, which include the blocking buffer, samples and antibodies, are all dispensed in separate droplets. Thus, Droparray is essentially a miniaturized version of singleplex ELISA, where each droplet region is treated as a separate well. The strength of our assay is that it uses a common sample (plasma) volume applied over all of the capture antibody spots in the panel, which minimizes the impacts of sample handling and pipetting errors that may occur with repeated pipetting into multiple separate wells.

In contrast to commercially available multiplex biomarker analysis systems, such as Luminex^TM^, ATPS-ELISAs can be performed with a broad range of detection antibodies, including polyclonal antibodies and cross-reacting antibodies. Luminex^TM^, on the other hand, must be carefully tested when introducing new antibody reagents, since it is not specifically designed to suppress antibody cross-reactivity. Moreover, our assay does not require specialized analyzers or sample handling devices. Instead, our assays can be read on common readers using plates that can be easily fabricated in many laboratories from bulk polystyrene by a hot-embossing-based rapid prototyping process. We envision such plates to become available for broader dissemination by injection molding. Finally, ATPS-ELISAs are highly scalable. By depositing antibody reagents in microdroplets, we prevent antibody cross-reactions and obtain multiplexed assays with comparable results to conventional singleplex ELISAs.

While many potential biomarkers have been discovered, there are multiple barriers (especially for multiplex biomarker assays) to biomarker verification, qualification and FDA approval. One technological barrier has been the lack of multiplex immunoassays that are efficient in usage of precious samples (consume less per run), robust in providing quantitative data without an increase of background or cross-reactions, rapidly customizable and free from reliance on difficult to access reagents, plates and hardware^32^,^35^. In addition, poor antibody specificity is problematic for immunoassays in general. Even with the best antibodies, high background and loss of linearity occurs with multiplexing due to higher total antibody concentrations. Our assay addresses these key areas. For example, the total concentration of detection antibodies in our system is low regardless of the degree of multiplexing, since all of the antibody solution droplets are separately spotted.

We also remove the bottleneck for clinical translation in several ways. First, multiplex signals become more robust due to lack of cross-reactions. Second, the validation procedure becomes easier because each biomarker in the panel is arrayed (or patterned) independently of the others. Therefore, if reagents change or one assay does not work, the remaining biomarkers are still valid. Finally, the increased variety of antibodies that can be used with ATPS-ELISA reduces the time and cost associated with the development and validation of new panels of multiplex immunoassays.

In the context of acute GVHD diagnosis and biomarker qualification, it is important to use a panel of multiple biomarkers^14^. Corticosteroids, which are general immunosuppressants, are used as the first line of treatment for patients presenting with GVHD symptoms, but fewer than half of patients treated with this standard therapeutic regimen have a sustained response^36^. Early identification of patients who will not respond to GVHD therapy is important because patients who develop steroid refractory acute GVHD have poor prognosis with an expected 1 year mortality of up to 90%^37^. Improving the risk-stratification of patients with GVHD using biomarker panels may permit early evaluation of additional therapies before the development of treatment-resistant disease. Equally important is the identification of patients who will respond well to treatment, which could allow for more rapid tapering of steroid regimens, thereby reducing toxicity such as increased risk for fatal infections or increased incidence of relapses due to impaired graft versus leukemia in low risk patients. Therefore, it is important to avoid false positive diagnoses that result from immunoassay cross-reactions.

The ability to identify high-risk patients using biomarker panels early in their transplant course, before the development of GVHD, may permit more stringent monitoring and preemptive interventions. Another avenue for using these biomarker panels is post-transplant monitoring of the response once the treatment has started^8^. Patients should be regularly monitored following bone marrow transplantation using non-invasive, rapid and standardized biomarker tests over the first month post-transplant, at the onset of acute GVHD clinical symptoms and throughout the GVHD treatment.

The compatibility of ATPS-ELISA with plasma suggests that our assay will also be compatible with other bio-fluids, such as urine, cerebral spinal fluid, salivary fluid and cell lysates. In addition, our test uses less sample, which is important for large retrospective studies where the volume of samples are often limited due to limited storage space. The judicious use of patient samples will also be relevant to pediatrics, were the volumes of samples are limited due to the size of children. Further studies with more rigorous standards for assay validation will enable translation to clinical biomarker qualification and determination of the clinical cutoffs for our assay^38^,^39^,^40^.

In conclusion, ATPS-ELISA provides multiplex biomarker detection for validation of new biomarker panels and diagnosis of complex diseases, such as GVHD, where custom multiplex immunoassays are required. The ATPS-ELISA technology prevents cross-reactions among antibody reagents, reducing the risk of false positive and false negative detection of disease markers. This strategy is compatible with standard immunoassay reagents, workflows and equipment, allowing it to be easily implemented in clinical and laboratory settings.

## Methods

### Reagents

ATPSs were created from aqueous solutions of 20 wt% DEX 500,000 (Pharmacosmos) and 20 wt% PEG 35,000. ELISA kits for human sTNFR1 (cat # DY225), human HGF (cat # DY294), human ST2 (cat # DY523) and human trappin-2/elafin (cat # DY1747) were purchased from R&D Systems. SuperSignal ELISA Femto Substrate (cat # 37074) was purchased from Thermo Scientific. Identical reagents, concentrations and incubation times were used for both the ATPS-ELISA and single ELISAs according to the manufacturer's recommendations.

### Partition Coefficients

The partition coefficients for the various biotinylated detection antibodies were determined by dot blot on polyvinylidene fluoride (PVDF) membranes. Briefly, the antibodies were diluted 1:500 and thoroughly mixed in ATPSs consisting of 10% PEG 35,000 and 10% DEX 500,000. The tubes were then centrifuged at 400 rcf for 15 min to cause complete phase separation of the PEG and DEX. The PEG and DEX fractions were collected and placed in separate tubes at 4°C. The PVDF membranes were prepared by soaking in methanol for 15 s, followed by soaking in water for 1 min and PBS for 5 min. The PVDF membranes were transferred onto PBS-moistened chromatography paper and spots containing 0.5 µL of the separated PEG and DEX samples were pipetted directly onto the membranes. The membranes were then blocked in 1% BSA for 1 h and washed 4 times in PBS containing 0.05% Tween-20. The membranes containing the PEG- and DEX-antibody spots were incubated in streptavidin-conjugated HRP for 1 h and washed 4 times with PBS. Chemiluminescence signals were developed using SuperSignal ELISA Femto Maximum Sensitivity Substrate (37074, Thermo Scientific) and detected using a Fluorchem M imager (Protein Simple).

### ELISA Protocol

Polystyrene plates were spotted with capture antibodies reconstituted in PBS (4 µL per spot). The plates were stored at 4°C overnight in sealed containers containing PBS-soaked Kimwipes to limit evaporation. The next day, the plates were washed five times with wash buffer (PBS, containing 0.05% Tween-20) and blocked with 3% casein for 1 h. After blocking, the plates were washed five times. The rapid and thorough washing performed in this step (and later after the detection antibodies were applied) ensured that the antibodies in solution would not be able to produce measurable cross-reactions with neighboring antibody spots. Next, the plates were incubated with the sample solutions (10 µL each for ATPS-ELISA) for 2 h. Following antigen application, plates were washed five times and incubated for 2 h with detection antibodies in either the traditional ELISA or ATPS-ELISA format. For conventional singleplex sandwich ELISA, detection antibodies were bath applied according to the manufacturer's specifications. For ATPS-ELISA, the plate was first filled with a solution of PBS containing PEG and 0.1% casein. DEX droplets containing the appropriate biotinylated detection antibodies (4 µL in volume) were then pipetted into the PEG over the corresponding capture antibody spots. In addition to providing phase-separation and confinement of the DEX droplets, the relatively large volume of PEG used to cover the plate prevented evaporation of the detection antibody solutions. The 4-plex antibody solutions were applied using a Matrix adjustable multipipettor (Thermo). An Ultimus I pneumatic pump (Nordson EFD) connected to a glass capillary needle containing the DEX/antibody solution was used to fill smaller wells (e.g., 9-plex assays or 16-plex assays). The details of this system and its operation are described elsewhere^41^,^42^. The plates were washed eight times followed by incubation with streptavidin-conjugated horseradish peroxidase for 1 h. Thorough and rapid wash buffer replacement ensured that there was no opportunity for detection antibody exposure to adjacent spots during washing. Plates were then washed five times and incubated with SuperSignal ELISA Femto Maximum Sensitivity Substrate (37074, Thermo Scientific). Chemiluminescent signal was detected using either a FluorChem M imaging system or a Synergy Neo HTS Multi-Mode Microplate Reader (Biotek). For single ELISAs the manufacturer's procedure was followed, with the exception that 384-well plates (rather than 96 well plates) were used with 10 µL assay volumes to conserve reagents.

### Plate fabrication

To optimize the plate design and demonstrate the capabilities of our multiplex system we fabricated a polystyrene ELISA plate using PDMS hot embossing. Xurography (razor writing) of ~110 µm-thick vinyl tape was used to generate tape templates for the larger sample wells and the smaller detection antibody wells^43^. The tape regions corresponding to the sample and antibody well regions were discarded and the sample well template was adhered to the antibody well template. The resulting double layer tape template was then adhered to a polystyrene dish. PDMS was cured above the tape template to form a reverse replica of the tape pattern that was used to emboss polystyrene to form the ELISA plate^44^. The final aluminum plate mold used to generate the plates for assay validation with the GVHD biomarkers was designed in AutoCAD (Autodesk) and SolidWorks (Dassault Systemes). An aluminum mold containing a 9 × 9 array of 6.5 mm diameter sample wells that each contained 4 1.5 mm diameter antibody wells was fabricated using precision CNC micromachining (Protomatic, Inc., Dexter, MI). Polystyrene dishes were heated above their glass transition temperature and the aluminum mold was stamped into the dish to mold the plate to its final dimensions. The 1.5 mm-deep antibody wells served three purposes. First, they held the capture antibody spots in place in the event of any unexpected plate movements during transport. Second, they provided a surface feature that could be detected visually and by contact with the pipette tips to align the DEX droplets containing the detection antibodies with the capture antibody patterns. Third, they prevented sliding of the DEX/detection antibody droplets during plate transport and incubation.

### Plasma samples

Heparinized plasma samples were collected from patients who received allogeneic bone marrow transplantation at the University of Michigan between 2000 and 2010. Plasma samples were collected under protocols approved by the University of Michigan Institutional Review Board and stored at the University of Michigan. The protein standard buffer consisted of PBS containing 10% healthy pooled plasma and 1% FBS, while sample buffer was PBS containing 1% FBS. Initial spike-and-recovery experiments indicated that signal intensities varied depending on patient plasma buffer and the sample matrix (plasma). To control for matrix effects, FBS was added to the PBS sample buffer such that appropriate signal intensities were achieve when control plasma was spiked with a known concentration of recombinant biomarker protein.

### Statistical analysis

All plots and statistical analyses were carried out in Sigmaplot with Sigmastat (Systat Software). Standard curves were generated over a wide range of concentrations to ensure that there were no hooking effects at high concentrations and to assess the level of non-specific background signal at low concentrations. The values for single ELISA (collected using a chemiluminescence plate reader) were scaled to produce values of the same order of magnitude as the ATPS-ELISA values (collected using the Fluorochem M western reader). The standard curves were fit using a four parameter logistic function. The limit of detection (LoD) was determined from the equation LoD = LoB + 1.645 (SDlow concentration sample), where SD is the standard deviation and LoB is the limit of blank^45^. LoB was calculated from LoB = meanblank + 1.645 (SDblank). Linear dynamic range (LDR) was determined using LDR = maximum linear response/LoD.

## Author Contributions

J.P.F. performed experiments, analyzed data, prepared the figures and wrote the manuscript. J.B.W. performed experiments, assisted with data analysis and figure preparation and helped write the manuscript. A.B.S. assisted with experiments and helped write the manuscript. M.T. assisted with experiments. S.P. and S.T. oversaw the project and the preparation of the manuscript. All authors reviewed the manuscript.

## Supplementary Material

Supplementary Information Supplemental Information
