# Supplementary material for: Aqueous two-phase system patterning of detection antibody solutions for cross-reaction-free multiplex ELISA
Source: Sci Rep. 2014 May 2;4:4878. doi: 10.1038/srep04878 (PMC4007081; doi:10.1038/srep04878)
Supplement: Supplementary Information — Supplemental Information [file srep04878-s1.doc]

Supplemental Information

Aqueous two-phase system patterning of detection antibody solutions for cross-reaction-free multiplex ELISA

John P. Frampton^a^, Joshua B. White^a^, Arlyne B. Simon^b^, Michael Tsuei^a^, Sophie Paczesny^c^* and Shuichi Takayama^a,b^*


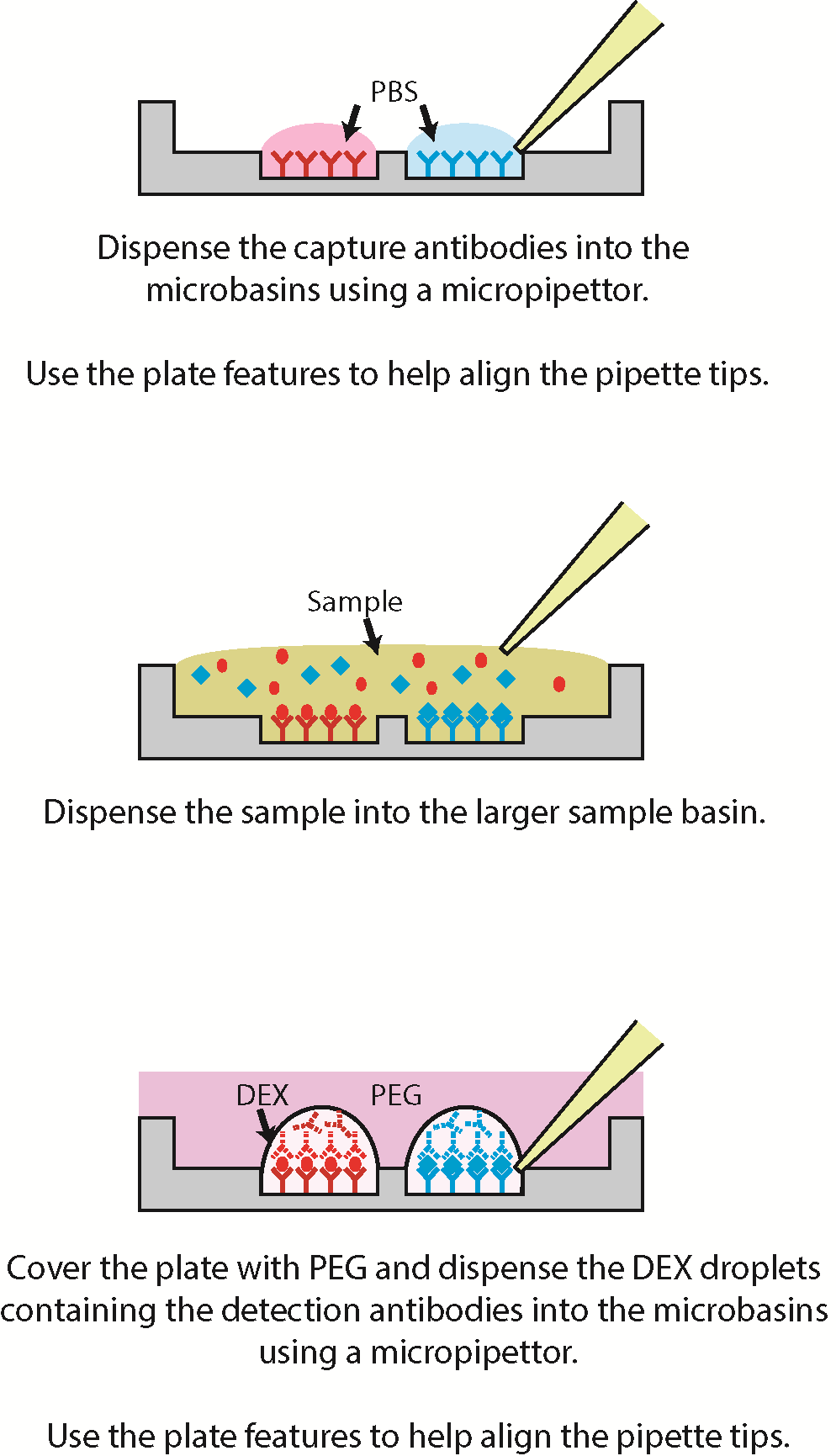


Figure S1: Schematic of capture antibody, sample and detection antibody alignment on embossed polystyrene plates.


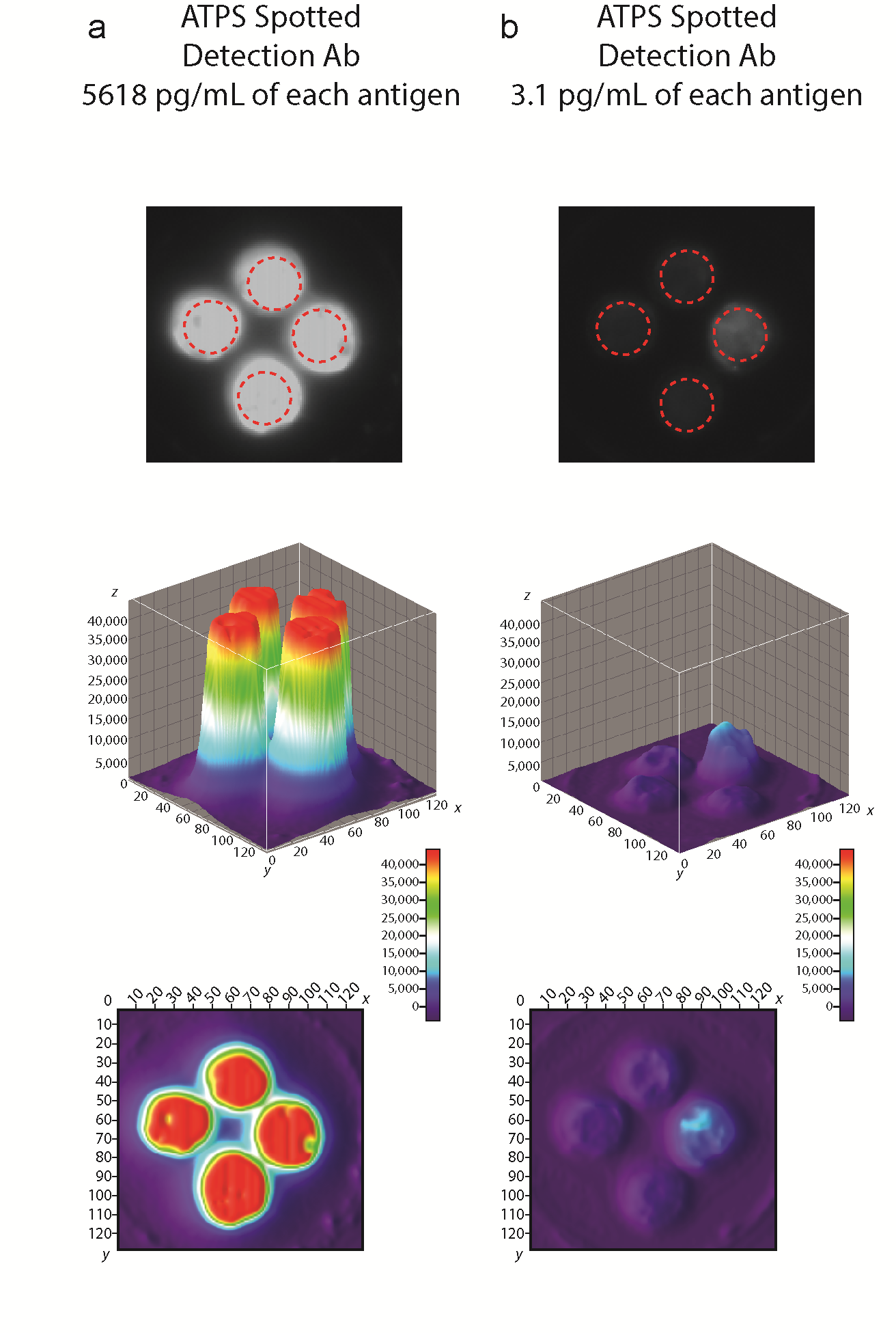


Figure S2: Analysis of optical crosstalk in the ATPS-ELISA system. (a) ATPS spotted detection antibodies with 5,618 pg/mL of each antigen. (b) ATPS spotted detection antibodies with 3.1 pg/mL of each antigen. Red dashed circles indicate sample areas that would be used for the densitometric analysis of the raw images. The extent of optical crosstalk was negligible (i.e., the pixel intensity at areas between spots approached background levels) for the areas sampled, as indicated by the heat maps for pixel intensity. Heat maps also demonstrated that the intensity of spots were relatively uniform with the ATPS spotting method for both high and low antigen concentrations. Heat maps were generated in ImageJ using the Interactive 3D Surface Plot plugin (Internationale Medieninformatik, Berlin, Germany).


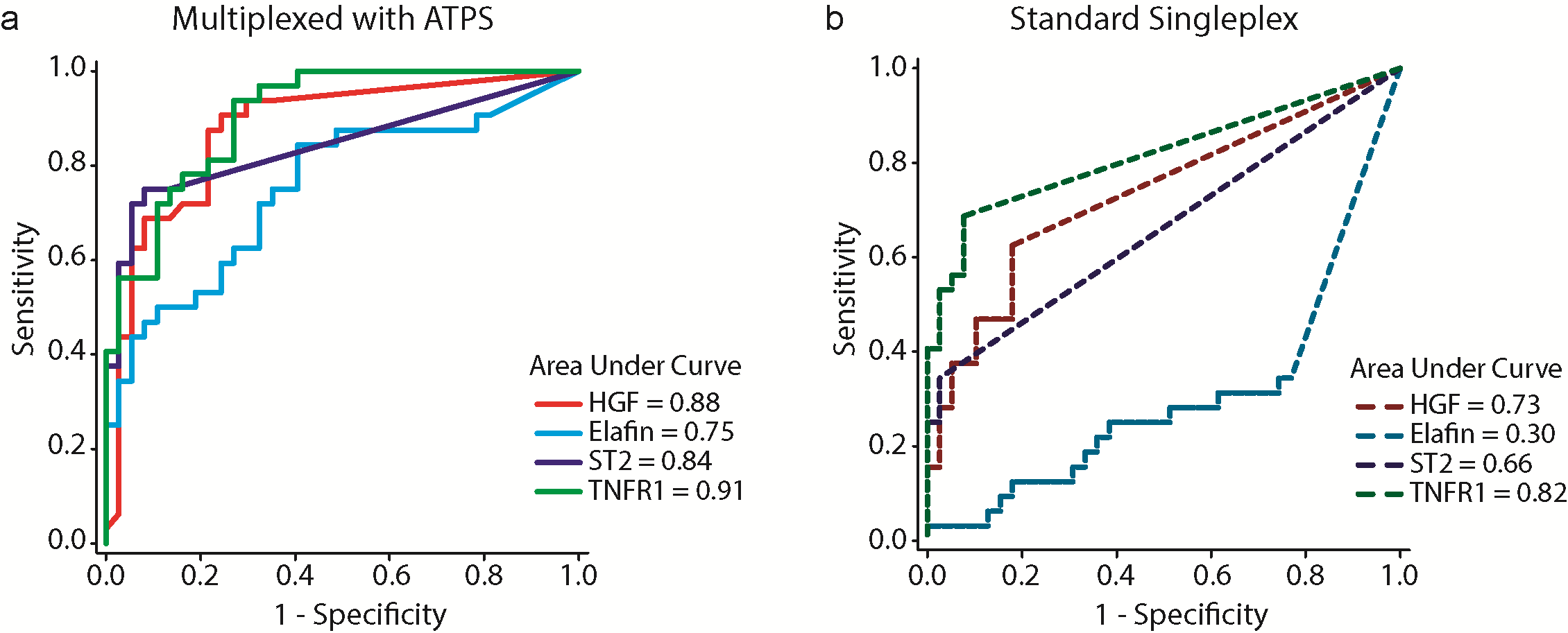


Figure S3: ROC analysis for ATPS-ELISA and individual (singleplex) ELISA. The curves for multiplex ATPS-ELISA (a, solid lines) demonstrate that the assay has high sensitivity and specificity for determining GVHD status from biomarker levels in patient plasma. Single sandwich ELISAs (b, dashed lines) were slightly less sensitive and specific. Larger studies beyond the scope of this study using additional samples and biomarkers, along with inter-user comparisons, will be required to conclusively verify these differences.
